# Lauroylated, Acetylated, and Succinylated *Agave tequilana* Fructans Fractions: Structural Characterization, Prebiotic, Antibacterial Activity and Their Effect on *Lactobacillus paracasei* under Gastrointestinal Conditions

**DOI:** 10.3390/polym15143115

**Published:** 2023-07-21

**Authors:** Dafne I. Díaz-Ramos, Rosa I. Ortiz-Basurto, Oscar García-Barradas, Martina A. Chacón-López, Efigenia Montalvo-González, Luz A. Pascual-Pineda, Uri Valenzuela-Vázquez, Maribel Jiménez-Fernández

**Affiliations:** 1 Laboratorio Integral de Investigación en Alimentos, Tecnológico Nacional de México-Instituto Tecnológico de Tepic, Tepic 63175, Nayarit, Mexico; daivdiazra@ittepic.edu.mx (D.I.D.-R.); mchacon@tepic.tecnm.mx (M.A.C.-L.); emontalvo@ittepic.edu.mx (E.M.-G.); 2 Instituto de Química Aplicada, Universidad Veracruzana, Xalapa 91190, Veracruz, Mexico; osgarcia@uv.mx; 3 Centro de Investigación y Desarrollo en Alimentos, Universidad Veracruzana, Xalapa 91190, Veracruz, Mexico; lpascual@uv.mx; 4 Agave Fructans Discovery Centre, Ayotlán 47930, Jalisco, Mexico; uvalenzuela@nutriagavesgroup.com

**Keywords:** fructans fractions, esterification, antibacterial activity, prebiotic potential, probiotic survival

## Abstract

The effect of chemical modification of fractions of native agave fructans (NAF), high performance (HPAF), and a high degree of polymerization (HDPAF) by lauroylation, acetylation, and succinylation reactions on their prebiotic activity, antibacterial properties were evaluated and survival of *L. paracasei* in a simulated gastrointestinal system. The characterization of the reactions was confirmed by NMR and FTIR. The lauroylated and succinylated fructan fractions showed higher antibacterial activity against pathogenic bacteria such as *Escherichia coli, Enterococcus faecalis* and *Staphylococcus aureus* than the unmodified ones. Analyses with *L. paracasei* showed that the acetylated fructan fractions had a greater prebiotic effect, and simulated gastrointestinal tests demonstrated that the acetylated and succinylated fractions favored the survival of *L. paracasei* during the gastrointestinal phase. The effect of modifying the agave fructans fractions on the evaluated properties depended on the structure, size, and polarity of each incorporated functional group, as well as the degree of polymerization and substitution of each fraction. These results show that the chemical modification of the fructan fractions analyzed improves their functional properties, offering an alternative in the food and pharmaceutical industry.

## 1. Introduction

Fructans are a mixture of fructooligosaccharides and fructose polymers linked by glycosidic bonds [1,2]. These can be obtained from different sources. Agave (*Agave tequilana Weber* var. azul) is an important fructan resource and possesses fructose polymers (units) with different degrees of polymerization, which according to the method by which they are obtained, can be classified into high-performance fractions (HPAF) (with reduction of simple sugars 5%), and a high degree of polymerization (HDPAF) (GPa > 30) [3], which have a different composition in relation to the number of fructoses and free sugars, which affect or influence their properties. There is evidence that fructans possess prebiotic activity as they contain β-type linkages that are not hydrolyzed by digestive enzymes but are fermented by lactic acid bacteria present in the intestinal microbiota [4]. These exhibit several properties that point to their use as important additives in food preparation [5]. However, they present limitations to being used in some food and pharmaceutical applications, so chemical modification may be an alternative to modify these properties and diversify their applications [5,6]. To date, several modification techniques are reported, including physical, chemical, and enzymatic [7]. Chemical modification through the lauroylation reaction of fructans has been shown to improve some of the stability properties of fructans, ensuring the protection and incorporation of compounds in the human organism [5,8]. In turn, it has been reported that the acetylation reaction improves the physicochemical and functional properties of starches and polysaccharides [9,10]. Similarly, the modification of biopolymers such as pullulan and starch by succinylation reaction has been investigated [11,12]. In general, the chemical modification of carbohydrates favors their affinity with various substances by increasing their hydrophobic nature and improving the functional properties at certain pHs [8,12,13], which would contribute to diversifying their application. Therefore, the present work has as its main purpose to evaluate the effect of chemical modification by esterification with lauroyl, acetyl, and succinyl groups of *Agave tequilana* native fructans fractions (NAF), and high performance (HPAF), and high degree of polymerization (HDPAF) on prebiotic, antibacterial properties, and *L. paracasei* survival in a simulated gastrointestinal system.

## 2. Materials and Methods

### 2.1. Materials

The native agave fructans (NAF) were donated by the national agave producer (Nutriagaves de México S.A. de C.V). The two agave fructan fractions, high-performance (HPAF) and a high degree of polymerization (HDPAF), were obtained according to Ceja-Medina et al. [3]. Lauroyl chloride #156930 (98% purity) and succinic anhydride #239690 (purity ≥ 99%) were obtained from Sigma Aldrich, (St. Louis, MO, USA), acetic anhydride #0655 (ACS purity, ≥ 97%) from Reactivos Química MEYER, (CDMX, Mexico) and N, N-dimethylformamide (DMF) # 03971 Fermont, (Monterrey, NL, Mexico). Broth and MRS agar (DifcoTM) were obtained from Thermo Fisher Scientific, MA, USA, (CDMX, Mexico) and BHI medium from MCD-LAB (Edo.de Mex., Mexico). Solutions were prepared with distilled water. Bacterial strains, *Staphylococcus aureus* (*S. aureus,* ATCC 25923), *Escherichia coli* (*E. coli*, ATCC 25922), *Salmonella typhi* (*S. typhi,* ATCC 14028) and *Enterococcus faecalis* (*E. faecalis*, ATCC 19433) were cultured at the Universidad Popular Autonoma del Estado de Puebla, Mexico.

### 2.2. Fructans Modification (Lauroylated, Acetylated, and Succinylated)

For the lauroylation reaction, 10 g of NAF, HPAF, or HDPAF were added to NaOH solution (50 mL, 20% *w*/*v*) at 25 °C under stirring, then 3 mL of lauroyl chloride was added dropwise and kept under stirring for 90 min. The resulting product was precipitated and filtered (5–11 µm) under a vacuum. Finally, the product was thermally refluxed for 8 h (by soxhlet, using hexane) to remove unreacted fatty acid chlorides. The product was dried under a vacuum at 60 °C for 1 h and recovered as a solid powder [14].

Acetylation was carried out by adding 1 g of NAF, HPAF or HDPAF in 10 mL of N, N-dimethylformamide (DMF) under stirring (300 rpm) until a uniform solution was obtained. Subsequently, sodium acetate (0.05 g) was added as a catalyst, and 6.6 mL of acetic anhydride was added to the reaction and kept at room temperature under stirring for 24 h. Afterward, the sample was precipitated with 50 mL of diethyl ether (Analytika Mexico, reagent grade) and water. The product was dried under vacuum at 60 °C and recovered as a solid powder [15].

Succinylation was performed by dissolving 1 g of NAF, HPAF or HDPAF in 12 mL of N, N-dimethylformamide (DMF) under stirring (300 rpm) until a uniform solution was obtained. Subsequently, sodium acetate (0.628 g) was added as a catalyst, and 4 g of succinic anhydride was incorporated into the reaction and kept at room temperature under stirring for 24 h. Subsequently, the sample was precipitated with 30 mL of diethyl ether (Analytika Mexico, reagent grade). The product was dried under vacuum at 60 °C and recovered as a viscous liquid to the touch [15].

### 2.3. Fourier Transform Attenuated Total Reflection Infrared Spectroscopy (FTIR-ATR)

IR spectra of unmodified NAF, HPAF, and HDPAF and their modified derivatives were analyzed by Fourier transform infrared spectroscopy using a Thermo Scientific Nicolet iS50 spectrograph coupled to an attenuated total reflectance diamond crystal (Thermo Electron Scientific Instruments LLC, Madison, WI, USA). Samples (2 mg) were placed directly into the apparatus without prior preparation. FTIR-ATR spectra were recorded with a resolution of 8 cm^−1^ in the spectral range of 4000−400 cm^−1^. Spectral analysis was performed with OMNIC version 8.2.0 software. (Thermo Fisher Scientific Inc., Waltham, MA, USA).

### 2.4. H^1^ NMR Spectroscopy

H^1^ NMR analysis for NAF, HPAF, and HDPAF unmodified and their modified derivatives was performed on a nuclear magnetic resonance spectrometer (Agilent Technologies, model DD2 500 MHz, CA, USA). Fifteen mg of sample and 500 μL of D_2_O were used as a solvent in the unmodified fructans. While in the lauroylated, acetylated, and succinylated samples, CD_3_OD, CD_3_COCD_3_ and D_2_O were used as solvents, respectively. Spectral analysis and visualization were performed with MestReNova (Mestrelab Research. Chemistry Software Solutions. Version. 12.0.0-20080.2.2.2.3). The degree of substitution (DS) of the modified fructans was calculated as the ratio between the area under the peak at 0.8–1.2 ppm divided by three (assigned to the methylene protons) and the area under the signal of the glucosidic proton of glucose [5].

### 2.5. Prebiotic-Viable Cell Count Assay

The prebiotic effect of the modified fructan fractions was performed according to the methodology described by Arrizon et al. [16] with modifications. Precultures were prepared by inoculating a 100 µL aliquot of *L. paracasei* in 10 mL of MRS broth (DifcoTM) and incubated at 37 °C for 11 h. Modified media were prepared, replacing dextrose (20 g/L) as a carbon source with lauroylated, acetylated, and succinylated agave fructans fractions, in addition to a positive control; 300 µL of each modified medium and the control were transferred to a 96-well plate in triplicate, containing 4.8 × l0^6^ CFU of pre-cultures with the microorganism. The optical density (OD) for each fraction was measured every hour at a wavelength of 560 nm, up to 40 h using a microplate reader (Spectrophotometer Multiskan Go, Thermo Fisher Scientific, Finland, USA), at a temperature of 37 °C with shaking before each measurement for 10 s. Subsequently, their viability was validated by plate counting in which modified media were prepared with unmodified and modified fructans (20 g/L), and the probiotic was allowed to grow in each modified medium (100 µL of *L. paracasei* in 10 mL of medium) for 11 h, then serial dilutions were made (10^6^), inoculated in Petri dishes with MRS agar, incubated for 24 h, and the colonies were counted.

### 2.6. Simulated GI Digestion of the Modified Fractions

Unmodified fructans and their derivatives were subjected to simulated GI digestion as described by Castro-Rodriguez et al. [17] with modifications. A pre-culture of *L. paracasei* strain was used by inoculating a 100 µL aliquot in 10 mL of MRS broth (DifcoTM), incubated at 37 °C for 11 h containing log 8.07 CFU/mL. Subsequently, 50 µL of the probiotic was taken, and 20 mg of each of the unmodified fructans and modified derivatives were added, respectively, to a 25 mL phosphate-buffered saline (PBS) volume and incubated for 24 h (solution 1). Simulated GI digestion consisting of the gastric and intestinal phases and simulated digestion fluids were prepared as follows: PBS was used as the stock solution for the fluids for a final volume of 25 mL for each fluid. The stock solutions for the simulated gastric fluid phase (SGF) consisted of 2.5 mL of solution 1 and 22.5 mL of pepsin (3 mg/mL) dissolved in PBS at pH 2.5 (solution 2) over a reaction time of 2 h. For the simulated intestinal fluid phase (SIF), 22.25 mL of pancreatin (1.9 mg/mL) dissolved in PBS at pH 6.5, 0.25 mL of bile salts (25 mg/mL) dissolved in 0.1 M sodium bicarbonate, and 2.5 mL of solution 2 were added at a reaction time of 2 and 5 h. Aliquots of each phase were taken to measure the viability of the probiotic in a solid medium. All digestion phases were carried out at 37 °C.

### 2.7. Antibacterial Activity (Microdilution Method)

Broth microdilution assays were evaluated as described by Mayrhofer et al. [18] with modifications using a microplate reader (Spectrophotometer Multiskan Go, Thermo Fisher Scientific, MA, USA, Finland). Cultures of pathogenic bacteria *(Enterococcus faecalis, Salmonella typhi, Staphylococcus aureus,* and *Escherichia coli*) were inoculated (1% *v*/*v*) in fresh medium, Brain Heart Infusion (BHI) for 24 h, and inoculated into 96-well plates (Costar-Corning Incorporated, Corning, NY, USA); 200 µL of test solution consisting of 100 µL of pathogenic culture and 100 µL of each fraction of unmodified fructans and modified derivatives were added to the wells. The plates were incubated at 37 °C for 24 h, and the growth of pathogenic bacteria was monitored by measuring the optical density (OD 600 nm). Antimicrobial activity was expressed as inhibition (%) of pathogen growth relative to the control (i.e., pathogen grown under optimal conditions, without sample).

### 2.8. Statistical Analysis

The experiments were performed twice with two replicates each. The means and the standard deviation of the means are calculated for each experimental parameter. The normality and distribution of the data were confirmed using the Shapiro−Wilk test. A one-way analysis of variance (ANOVA) and a pairwise comparisons test (Holm−Sidak test) were performed to identify significant differences with a significance value α < 0.05 [19]. All statistical procedures were performed with the SigmaPlot version 12.0 software (Copyright © 2011 systat software, Inc. Munich, Germany). For the graphic representation, Kaleida Graph version 4.0 was obtained.

## 3. Results

### 3.1. Characterization by FTIR and ^1^H NMR

**Figure 1:**
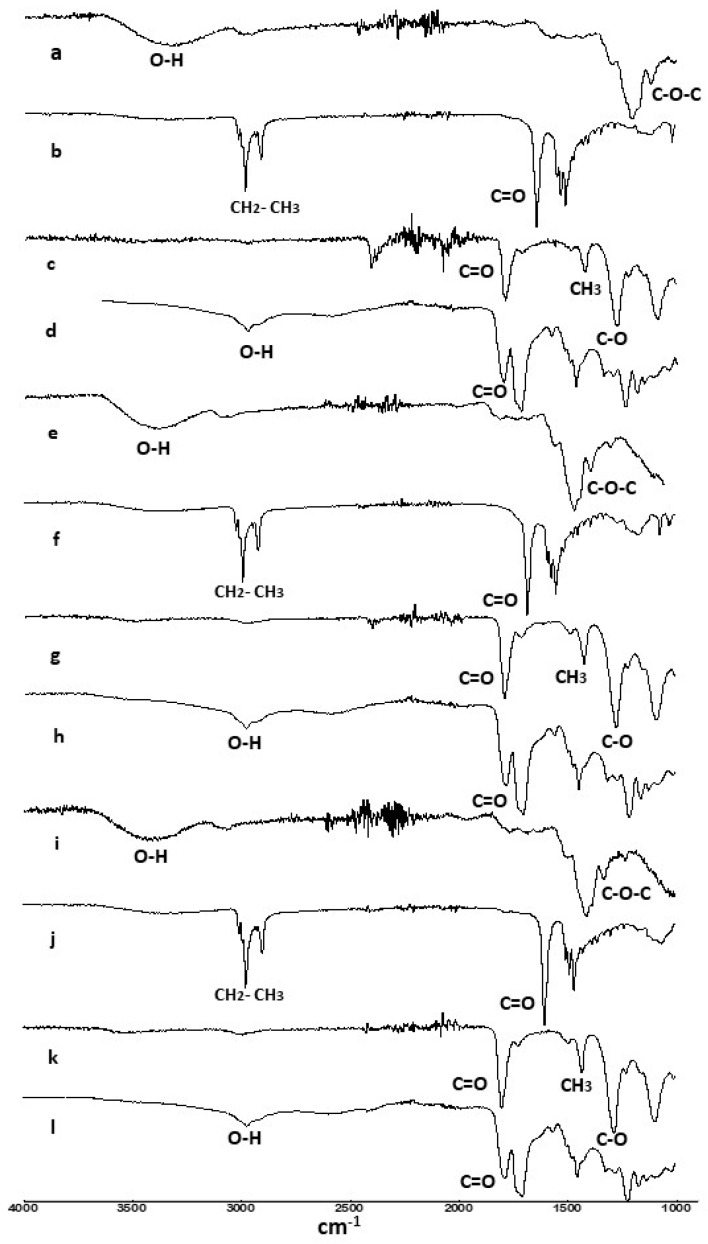
FTIR spectra of unmodified NAF (**a**), lauroylated (**b**), acetylated (**c**), succinylated (**d**), un-odified HPAF (**e**), lauroylated (**f**), acetylated (**g**), succinylated (**h**) and unmodified HDPAF (**i**), laurylated (**j**), acetylated (**k**), succinylated (**l**).

**Figure 2:**
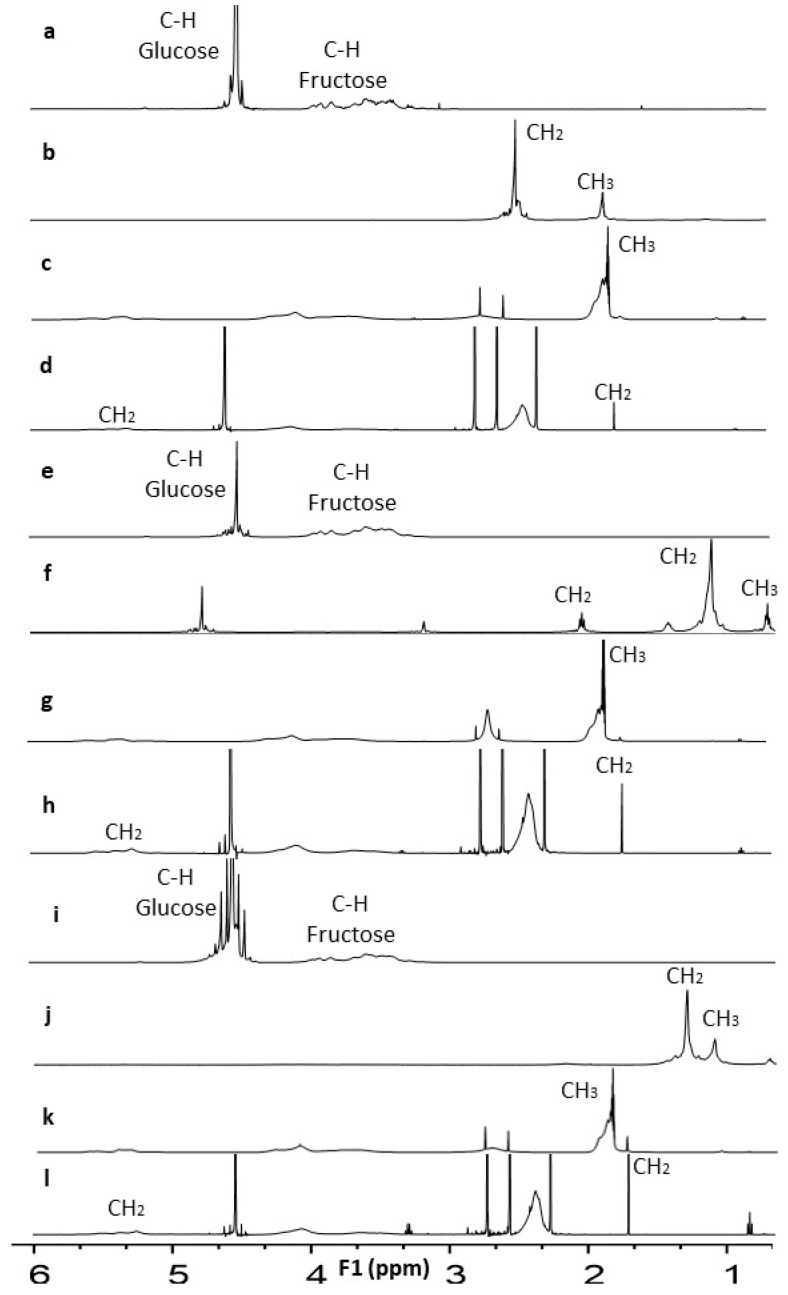
NMR spectra of unmodified NAF (**a**), lauroylated (**b**), acetylated (**c**), succinylated (**d**), unmodified HPAF (**e**), lauroylated (**f**), acetylated (**g**), succinylated (**h**) and unmodified HDPAF (**i**), lauroylated (**j**), acetylated (**k**), succinylated (**l**).

The unmodified and lauroylated, acetylated, and succinylated agave fructans fractions were analyzed by FTIR and ^1^H NMR (Figure 1 and Figure 2). The FTIR spectra of the unmodified fractions present stretching bands at 3268 and 3274 cm^−1^ and 1032 and 1012 cm^−1^, for NAF, HPAF, and HDPAF, respectively, which are characteristic of the -OH and C-O-C groups. In general, the modified fructans showed a shift in the main bands. The lauroylated fructans showed new absorption bands at 2921 and 2847 cm^−1^, while the acetylated fractions exhibited peaks at 1739.82 cm^−1^, as well as a -CH_3_ bending peak at 1368 cm^−1^. In turn, the succinylated fractions presented new bands at 2931 cm^−1^ corresponding to the -OH stretching vibrations of the carboxylic acid formed, in addition to a signal at 1643 cm^−1^ attributed to the carbonyl group (C=O) of the same acid, as well as a peak at 1724 cm^−1^ corresponding to the carbonyl group (C=O) of the ester formed [20,21]. In the succinylated fractions the succinic anhydride forms two carbonyl groups, due to the formation of carboxylic acid, while in the acetylated fractions this band does not appear, but they present a signal that corresponds to the bending of the methyl (CH_3_) attached to the acetyl group at 1368.23 cm^−1^ as well as the one emitted by C-O vibrations at 1219.84 cm^−1^. The efficiency of the reaction was evaluated by analyzing the degree of substitution. Figure S1 shows the reaction mechanism of Agavin, which is the main component of agave fructans. The laurylated (a), acetylated (b), and succinylated (c) fractions were prepared by an esterification reaction. The laurylated samples presented degrees of substitution (DS) from 2.03 to 2.36, while the acetylated samples showed from 2.61 to 2.78 and the succinylated from 1.91 to 2.0. Some studies have reported similar DS [5] and others with lower DS [13,22,23] than those found in the present work; these differences are attributed to factors such as DP, type, and method of modification used (reaction conditions).

The chemical modification of fructans and their fractions by laurylation, acetylation, and succinylation was corroborated by ^1^H NMR (Figure 2). The unmodified fructans presented signals at 3.65, 3.52, and 4.08 ppm corresponding to the fructose proton. At 4.66, 4.64, and 4.77 ppm, they are attributed to the anomeric proton of glucose, for NAF, HPAF, and HDPAF, respectively.

### 3.2. Solubility of Agave Fructans Fractions Modified

**Figure 3:**
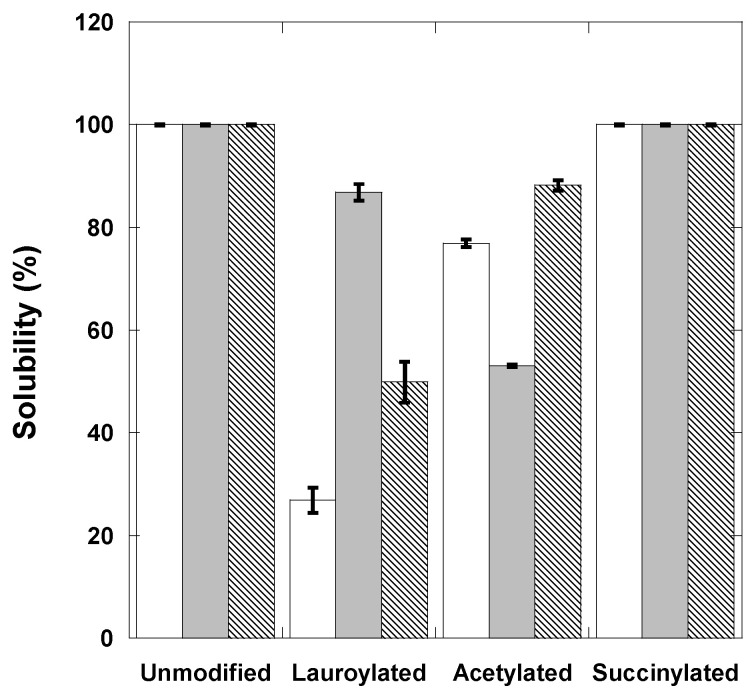
Solubility of fructans NAF (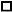), HPAF (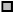) and HDPAF (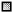) unmodified and modified.

The native fructans and their lauroylated and acetylated fractions significantly decreased the solubility in water, which was dependent on the fraction used, presenting differences (*p* < 0.05) among them (Figure 3). The water solubility of the lauroylated samples decreased after the modification, being the samples of native fructans modified by lauroylation were those that exhibited the lowest solubility in water (26.90%), followed by HDPAF (49.90%) and HPAF (86.90%).

### 3.3. Prebiotic Activity of Modified Agave Fructans Fractions

**Figure 4:**
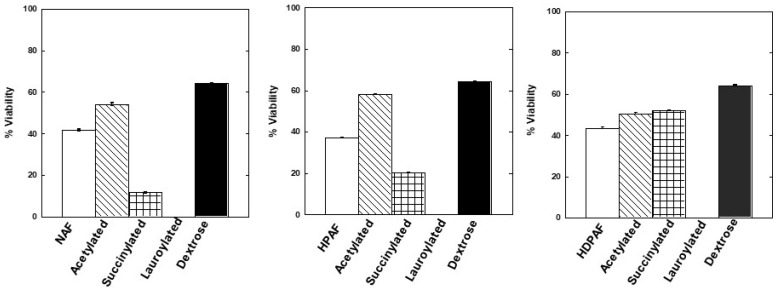
Viability of *L. paracasei* in enriched media with NAF, HPAF, HDPAF, unmodified and modified.

Figure 4 shows the viability of *Lactobacillus paracasei* when the carbon source was replaced by acetylated and succinylated (Supplementary Materials Figure S2) fructans fractions compared to the control treatments (no modification and dextrose as carbon source). Lauroylated fructans fractions are not presented in the figure as they did not show viability, which could be due to the fact that lauric acid incorporated in this reaction confers antimicrobial properties [24]. The viability of *Lactobacillus paracasei* was higher in media with acetylated fructans fractions (50.6–58.26%) compared to unmodified fractions (37.33–43.56%).

### 3.4. Antibacterial Activity of the Modified Fructans Fractions

**Figure 5:**
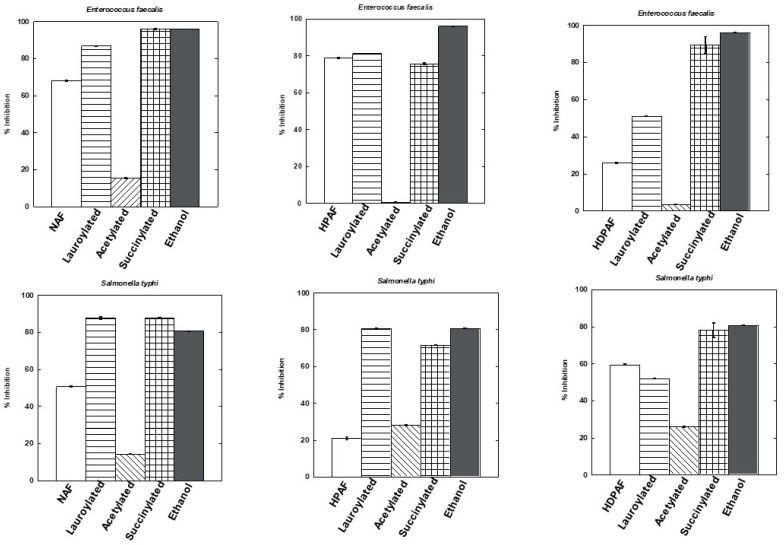

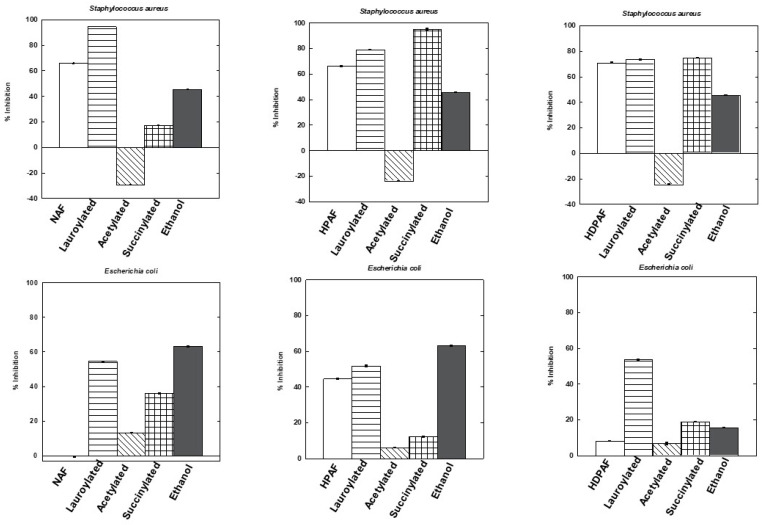
Percent inhibition of unmodified and modified fructans (10 mg/mL) against *Enterococcus faecalis, Salmonella typhi, Staphylococcus aureus,* and *Escherichia coli*.

The antibacterial activity of the modified agave fructans fractions against four pathogenic bacteria (*Enterococcus faecalis, Salmonella typhi, Staphylococcus aureus*, and *Escherichia coli*) was investigated. Figure 5 shows that the modified NAF, HPAF, and HDPAF exerted a greater inhibitory effect against pathogenic bacteria than was shown by the unmodified ones. The unmodified HPAF fraction showed greater antimicrobial activity against *E. faecalis* (79.00%) and *S. aureus* (66.24%), and the unmodified HDPAF fraction showed greater inhibition (70.83%) against *S. aureus*. It is important to highlight that the activity of fructans modified by the different reactions was dependent on each bacterium. In general, lauroylated fructans and succinylated fructans exhibited higher antimicrobial activity against the test bacteria compared to acetylated fructans. The lauroylated fractions showed inhibition ranges of 51–94.58%, the succinylated fractions of 36.05–95.94%, and the acetylated fractions of 3–26%. In contrast, the acetylated fractions showed growth of *Staphylococcus aureus*, possibly because the acetyl groups are metabolized by this microorganism [5,19].

### 3.5. Effect of Modified fructans on the Survival of Lactobacillus Paracasei under Simulated Gastrointestinal Conditions

**Figure 6:**
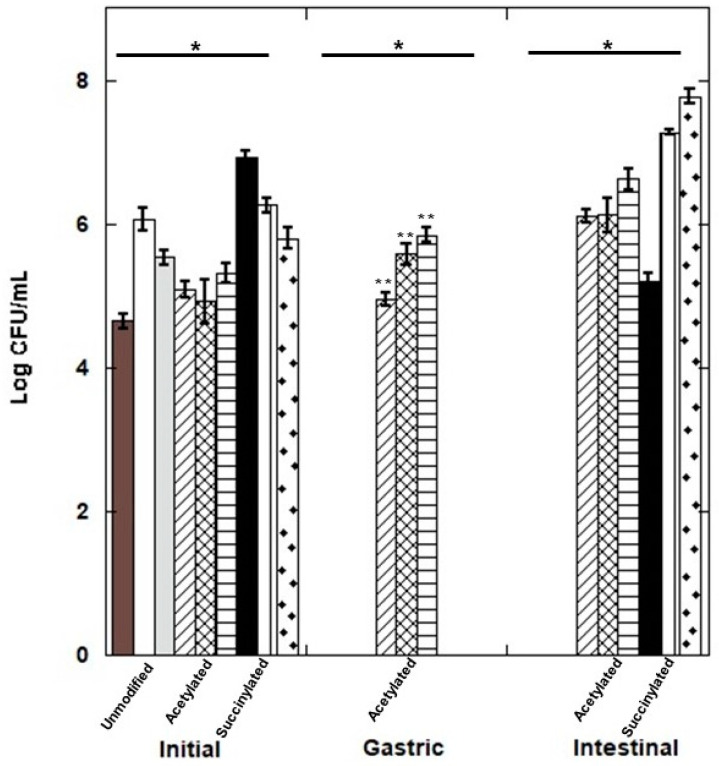
Survival of *L. paracasei* after passage through gastric and intestinal conditions. Log CFU/mL: colony forming units per milliliter. NAF (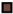), HPAF (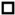), HDPAF (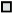) unmodified, NAF (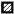), HPAF (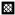), HDPAF (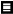), acetylated and NAF (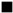), HPAF (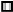), HDPAF (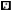) succinylated. Initial bacterial counts are shown in dashed bars (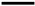). * *p* < 0.05 vs initial bacterial count; ** *p* < 0.05 vs gastric conditions.

The effect of modified fructan fractions (lauroylated, acetylated and succinylated) was evaluated on the survival of *Lactobacillus paracasei* under simulated gastrointestinal conditions. The lauroylated fractions did not show viability in any stages evaluated, so they were not included in this study. Figure 6 shows that *Lactobacillus paracasei* had a viability of 4.95, 5.58, and 5.84 log CFU/mL in the gastric phase for NAF, HPAF and HDPAF acetylated, respectively. In the intestinal phase, it was 6.10, 6.12, and 5.62 log CFU/mL for NAF, HPAF and HDPAF acetylated, respectively. For their part, the succinylated fractions presented values lower than 1 × 10^3^ CFU/mL (Supplementary Materials Table S1).

## 4. Discussion

The lauroylation was carried out by an addition/elimination reaction, in which the alkaline medium (NaOH) produces the deprotonation of the -OH groups in the fructans, causing the nucleophilic attack on the carbonyl group of the lauroyl chloride, forming an ester group and eliminating water and sodium chloride. As for acetylation and succinylation, the reactions occur through a nucleophilic attack. First, sodium acetate promotes the deprotonation of the -OH groups of the fructans to attack one of the carbonyl groups of the acetic or succinic anhydrides. It also acts as a catalyst by increasing the carbonyl activity by protonation of one of the carbons of the anhydrides. Subsequently, the nucleophilic attack of the oxygen of the -OH groups of the fructan on one of the carbons of the anhydrides takes place, forming the ester in each reaction and eliminating acetic acid and succinic acid in each one.

The new absorption bands present in the lauroylated fructans of the FTIR spectra demonstrated changes in their structure due to the symmetric and asymmetric stretching of the C-H bonds in the methyls and methylenes of the hydrocarbon chains, as well as the presence of the carbonyl group, found at 1557 cm^−1^, corresponding to the ester formation in the fructans [8,25], while the bands present in the acetylated fractions corroborated the presence of acetyl groups (-COCH_3_) demonstrating the esterification [26]. The differences in the functional groups are given by the group’s structure incorporated into different fructan fractions. In addition, a partial or total decrease in the intensity of the -OH bands are observed with the incorporation of the lauroyl, acetyl, and succinyl groups, which in turn is related to the degree of substitution.

NMR analysis revealed that the lauroylated fractions presented new signals at 2.05 and 2.65 ppm (NAF), 0.89, 1.28 and 2.19 ppm (HPAF), and 1.28 and 1.47 ppm (HDPAF), which are characteristic of the methyl and methylene groups of the incorporated fatty acid chain. The signals found in FTIR and ^1^H NMR for lauroylated, acetylated, and succinylated fructans agree with those reported for native fructans, inulins, and other chemically modified polysaccharides [5,8,11,12,27].

The modification of the solubility in water of the lauroylated and acetylated fractions is explained by the incorporation of lauroyl and acetyl groups which react with -OH groups to form hydrophobic esters, modifying their polarity, solubility and degree of substitution (DS) [22,28]. While in succinylated fructans, it is observed that solubility was not affected; this could be because succinylation increases the solubility indices by increasing its net negative charge, which causes a more significant interaction with water and, therefore, increases solubility [29]. In addition, substituting succinyl and carboxyl groups in the fructan molecule also increases solubility, which is related to the degree of succinylation [30].

The prebiotic activity of the modified fractions was evaluated by the growth capacity of *Lactobacillus paracasei*. Analysis of the viability of *Lactobacillus paracasei* in growth media with acetylated and succinylated fractions suggests that modification by acetylation favors microbial growth, which could be due to the availability of substrate due to the presence of fructanases and that of small acetyl groups that allow enzymes a better interaction with the substrate [5]. Similar results were reported for the fermentation of acetylated fructans with *Saccharomyces boulardii* because they can be used as a carbon source [19]. The differences between fructan fractions and their modified derivatives could be due to differences in the degree of polymerization and the chemical structure and fermentable carbohydrate content of each fraction [31].

The antibacterial activity effect of the modified fructans fractions against four pathogenic bacteria (*Enterococcus faecalis*, *Salmonella typhi*, *Staphylococcus aureus*, and *Escherichia coli*) could be due to the osmotic effect that causes a membrane permeability of the bacteria and prevents the entry of nutrients into the microorganism [32,33,34]. The differences between the fractions (*p* < 0.05) are influenced by their structure and composition, as it has been reported that higher molecular weight usually leads to lower inhibitory action [33]. The lauroylated and succinylated fractions exhibited greater inhibition of pathogenic bacteria, suggesting that they can be used as microbiological control agents.

The viability of *Lactobacillus paracasei* in the presence of fractions modified during passage through a simulated gastrointestinal system may be due to the adaptive capacity of the *Lactobacillus paracasei* to resist the harmful action caused by the bile in the intestine [35]. It has been reported that in *Lactobacillus paracasei* strains isolated from kefir, the proteins that are regulated following gastrointestinal stress are those involved in carbohydrate metabolism, focusing on the modification of isoforms of the cytoplasmic enzyme glyceraldehyde-3-phosphate dehydrogenase (GAPDH) and the biosynthesis of exopolysaccharides (EPS). Thus an increase in adhesion capacity could be attributed to factors such as increased EPS production and/or the presence of proteins that switch between unrelated functions depending on cell location [35]. The modified fractions allowed the increase in viability of the probiotic by allowing colonization of the intestine under gastrointestinal stress since, after the gastrointestinal passage, the acetylated and succinylated HPAF and HDPAF showed viability around 6–7 log CFU/mL, which is within the recommended range to favor colonization and have a therapeutic or functional effect in the intestine [36,37].

## 5. Conclusions

The characterization of the native agave fructans and their fractions by NMR and FTIR confirm their chemical modification by lauroylation, acetylation, and succinylation. The lauroylation and acetylation reaction significantly decreased the solubility of the agave fructans fractions, resulting in the production of hydrophobic fructans fractions. The acetylated and succinylated agave fructans fractions presented prebiotic activity and increased the survival of *L. paracasei* in the different stages of a simulated gastrointestinal system. The lauroylated and succinylated fractions presented a higher percentage of inhibition against pathogenic bacteria. The effects are related to the chain length, the degree of substitution obtained from the incorporated group, and the fructans polymerization degree. Therefore, chemical modifications are a choice for improving the functionality and properties of agave fructans (natives and fractions), increasing their value, thus diversifying their applications as food preservatives, antiseptics, prebiotics and probiotics, in diverse areas of industry. It is important to emphasize that it is necessary to carry out research in vivo for this type of product before defining its application in the human body.

## Data Availability

The data underlying this article are available in the article and in its online supplementary material.

## Supplementary Materials

The following supporting information can be downloaded at: https://www.mdpi.com/article/10.3390/polym15143115/s1, Figure S1: Proposed reaction mechanism of Agavin for the lauroylation (a), acetylation (b), and succinylated (c) reactions. Figure S2. Growth curves of NAF (a), HPAF (b), and HDPAF (c) unmodified (), acetylated, () and succinylated (). Figure S3. Full ^1^H NMR spectra. Table S1. Absorbance during gastric and intestinal simulation in succinylated fructans.
