# Supplementary material for: Lauroylated, Acetylated, and Succinylated Agave tequilana Fructans Fractions: Structural Characterization, Prebiotic, Antibacterial Activity and Their Effect on Lactobacillus paracasei under Gastrointestinal Conditions
Source: Polymers (Basel). 2023 Jul 21;15(14):3115. doi: 10.3390/polym15143115 (PMC10383541; doi:10.3390/polym15143115)
Supplement: Supplementary file 1 [file polymers-15-03115-s001.zip › polymers-2448428-supplementary.pdf]

Supplementary Materials:

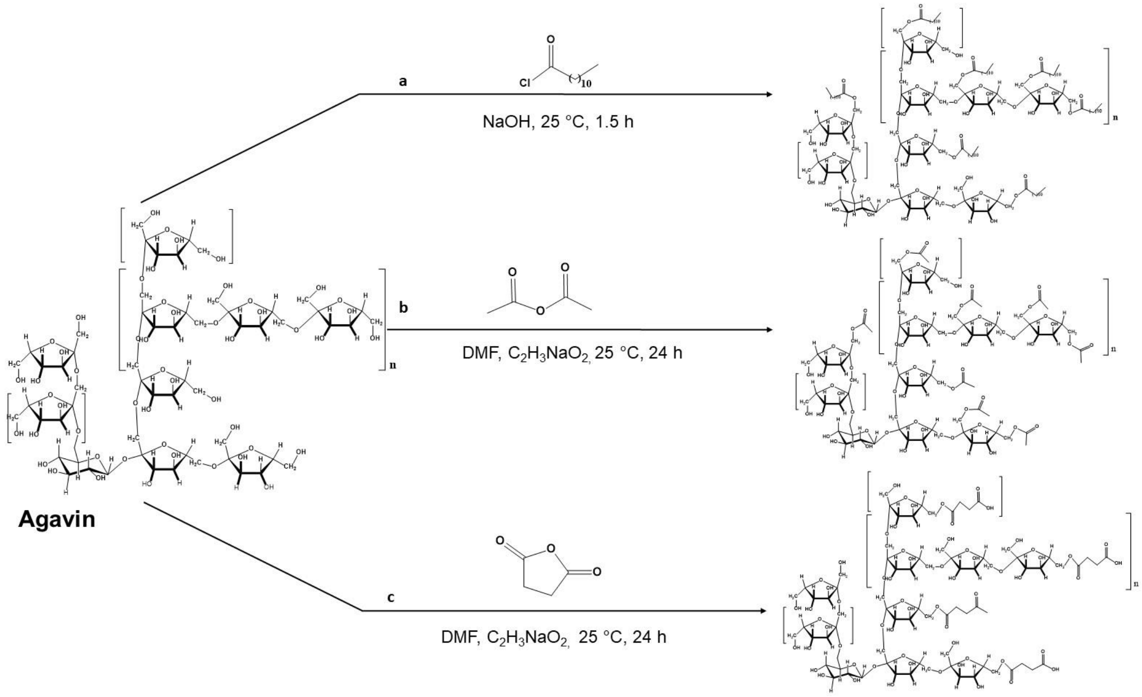

Figure S1. Proposed reaction mechanism of Agavin for the lauroylation (a), acetylation (b), and succinylated (c) reactions.

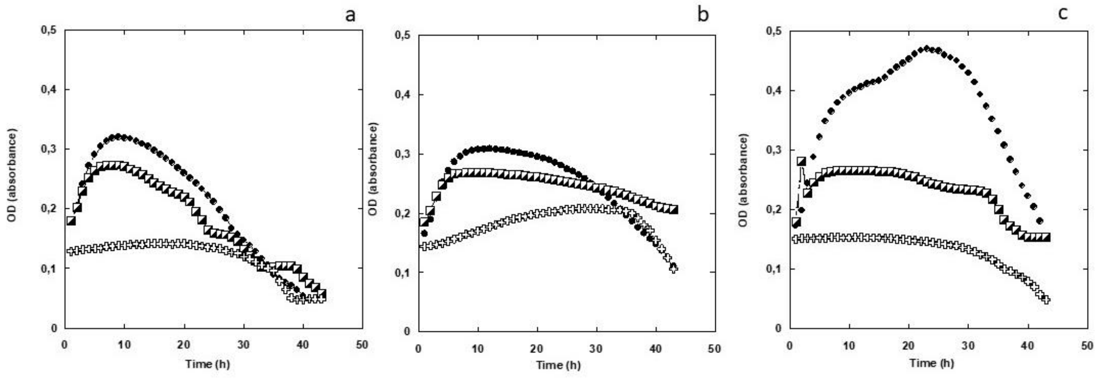

Figure S2. Growth curves of NAF (a), HPAF (b), and HDPAF (C) unmodified (●), acetylated, (-◪-) and succinylated (-✚-)

Table S1. Absorbance during gastric and intestinal simulation in succinylated fructans.

Fructans succinylated	Phase	Abs
**NAF**	Gastric	0.006 ± 0.01[a]
**HPAF**		0.003 ± 0.01[a]
**HDPAF**		0.002 ± 0.01[a]
	Intestinal	
**NAF**		0.022 ± 0.06[b]
**HPAF**		0.013 ± 0.01[b]
**HDPAF**		0.016 ± 0.01[b]

Values are given as mean ± SD (n = 3). Different letters denote significant differences (P < 0.05).

Figure S3. Full ^{1}H NMR spectra

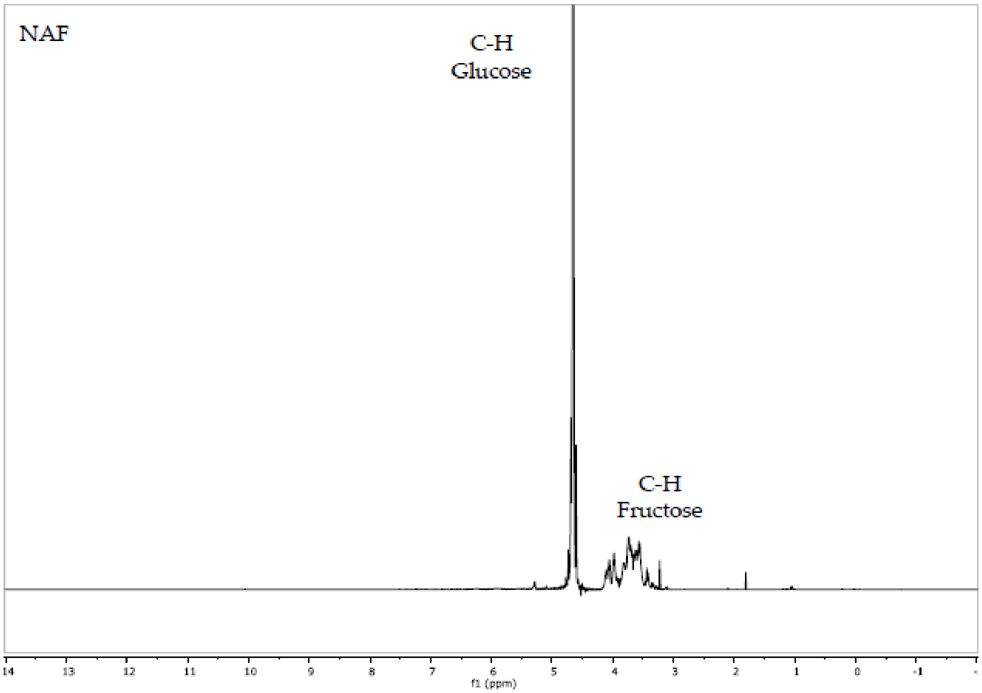

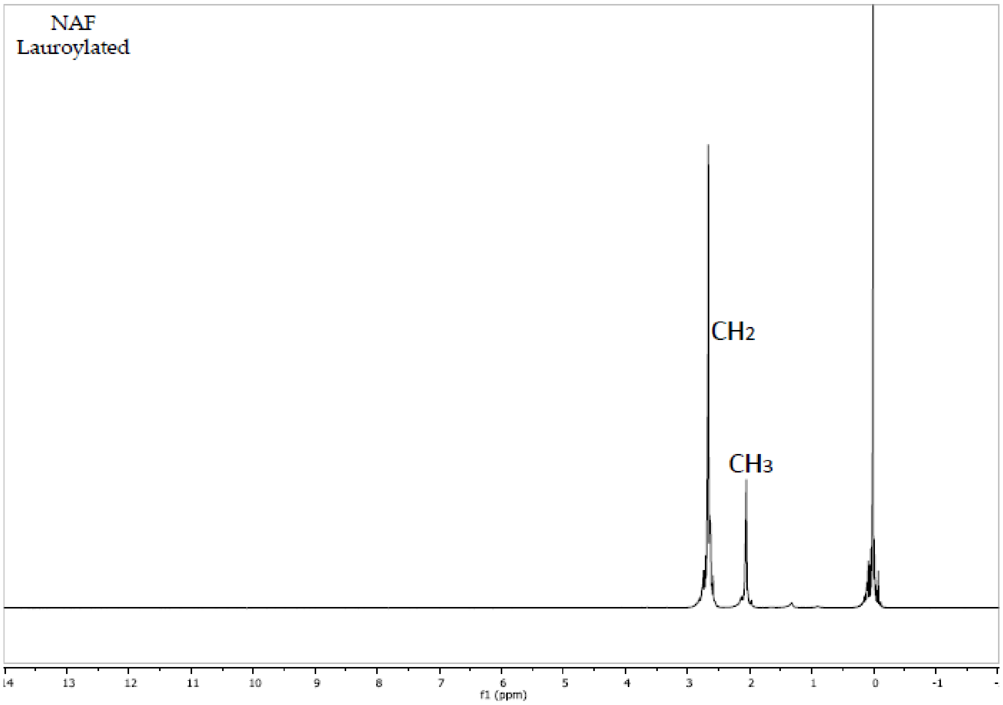
NAF
Lauroylated
CH2
CH3
14 13 12 11 10 9 8 7 6 5 4 3 2 1 0 -1
f1 (ppm)

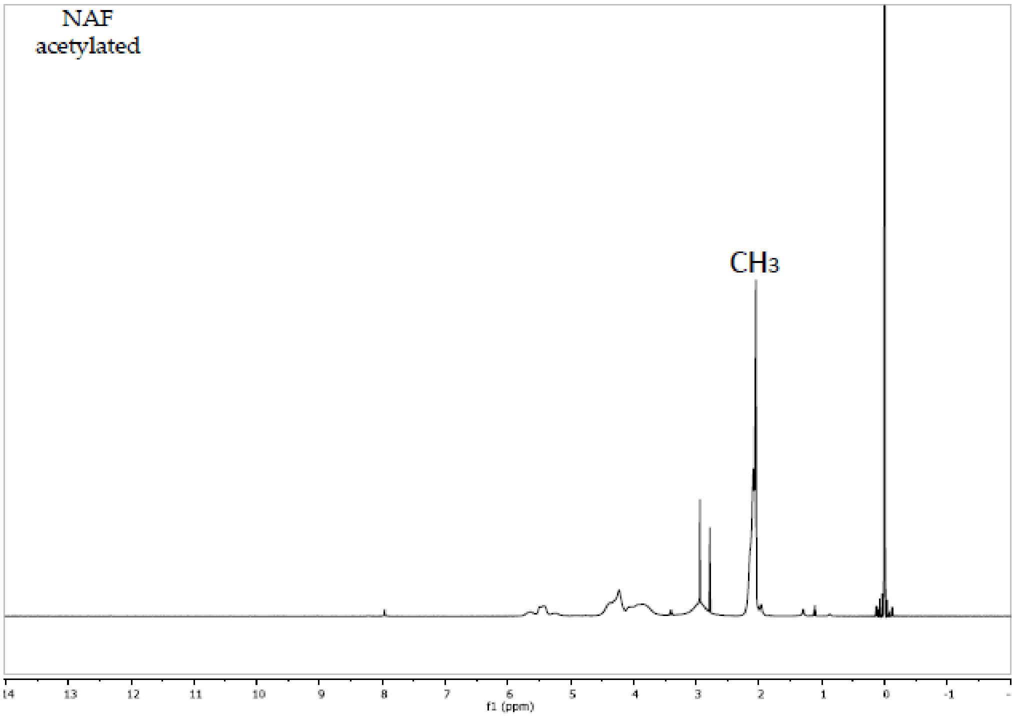
NAF
acetylated
CH3
14 13 12 11 10 9 8 7 6 5 4 3 2 1 0 -1
f1 (ppm)

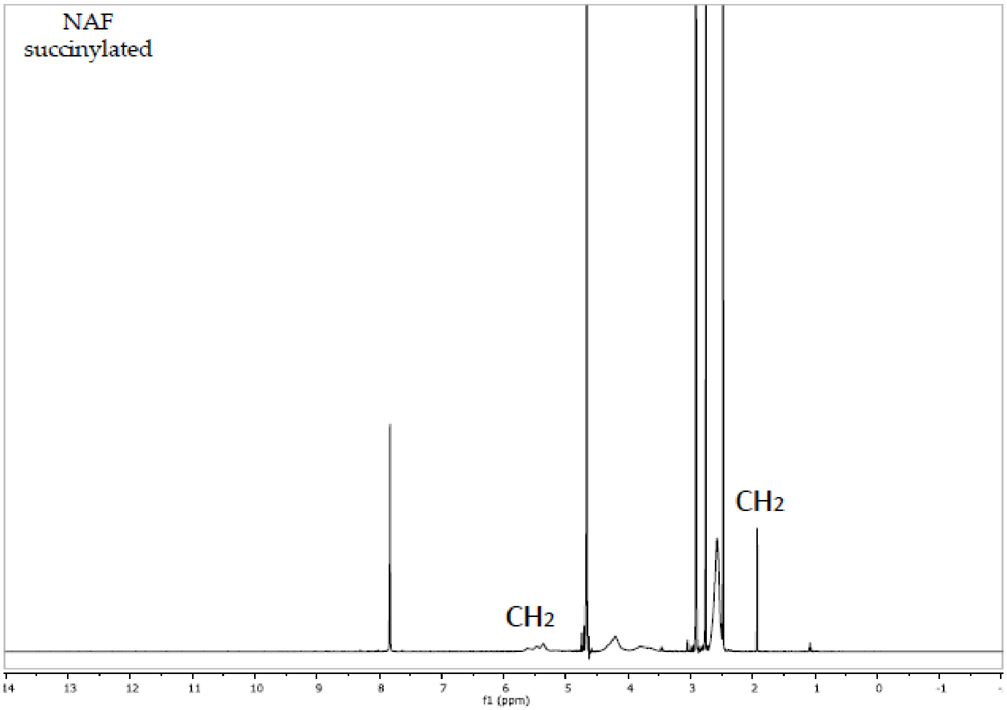
NAF
succinylated
CH2
CH2
f1 (ppm)

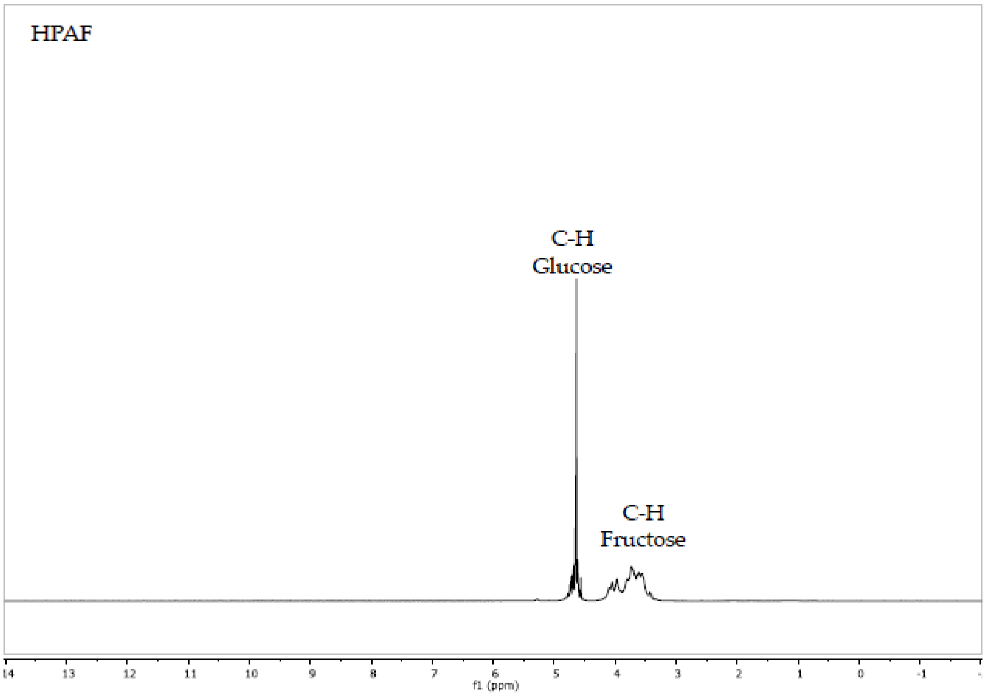
HPAF
C-H
Glucose
C-H
Fructose
f1 (ppm)

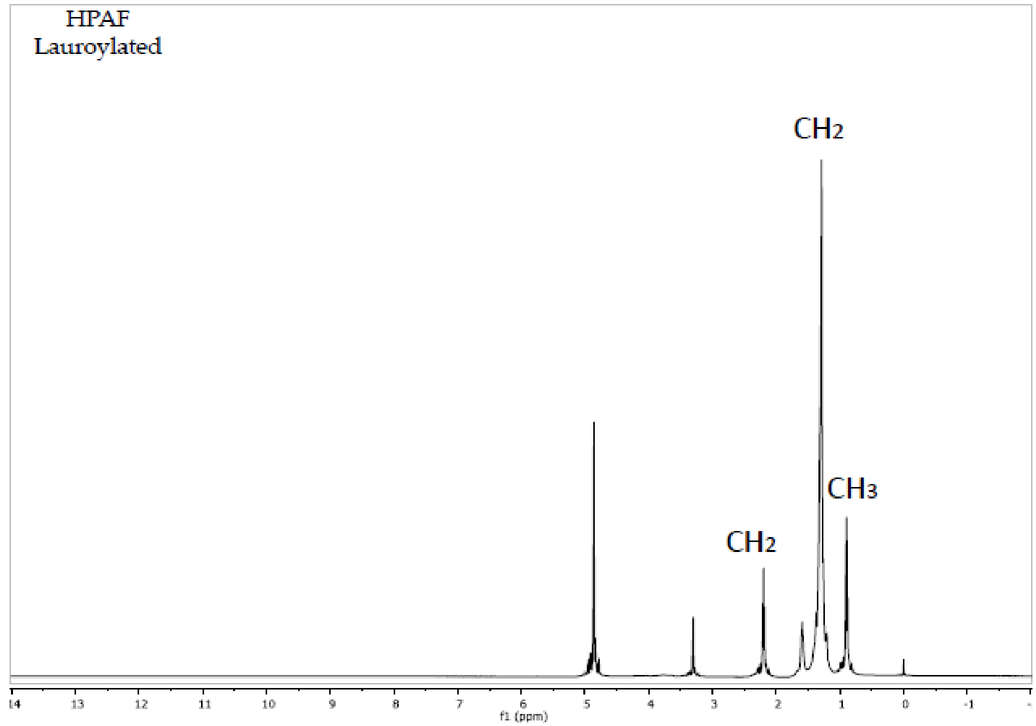
HPAF
Lauroylated
CH2
CH3
CH2
f1 (ppm)

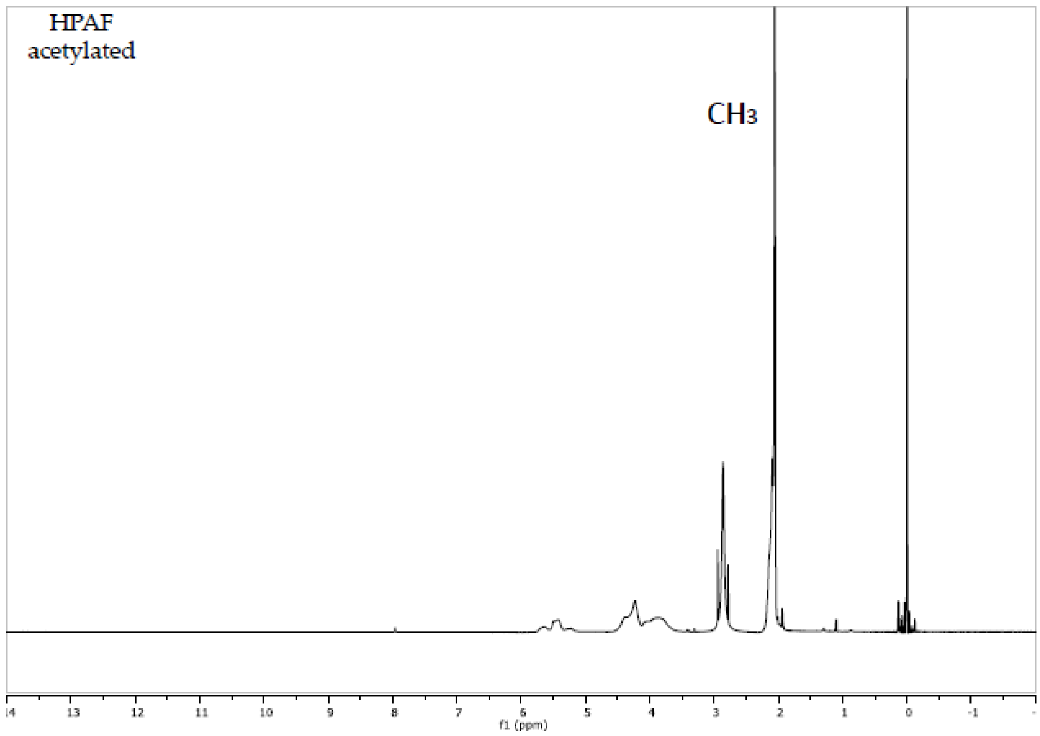
HPAF
acetylated
CH3
f1 (ppm)

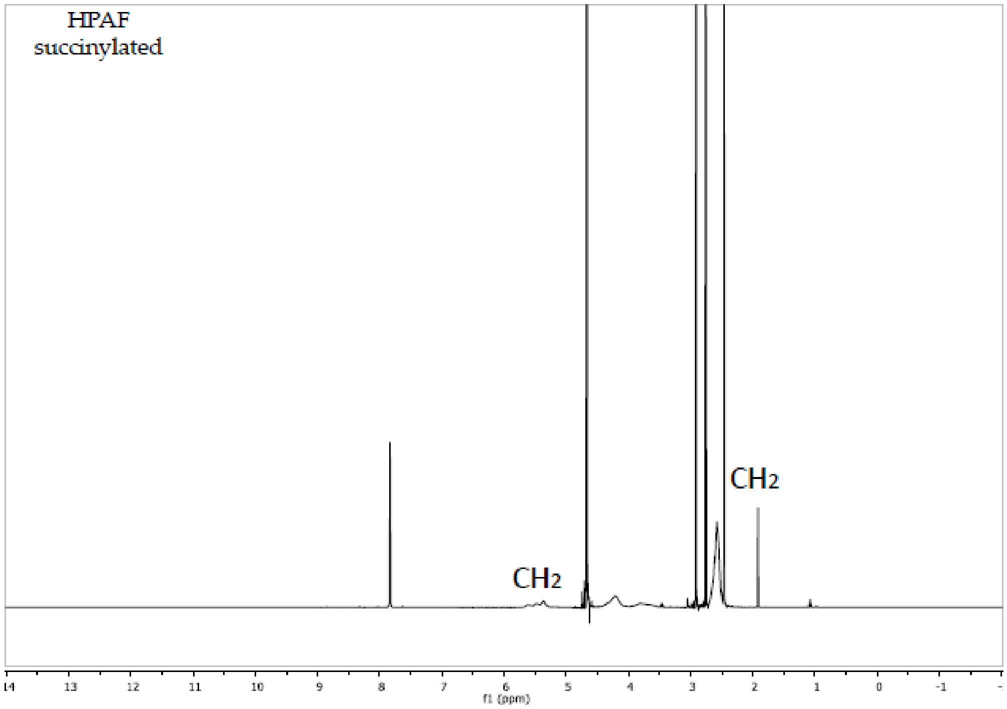
HPAF
succinylated
CH2
CH2
f1 (ppm)

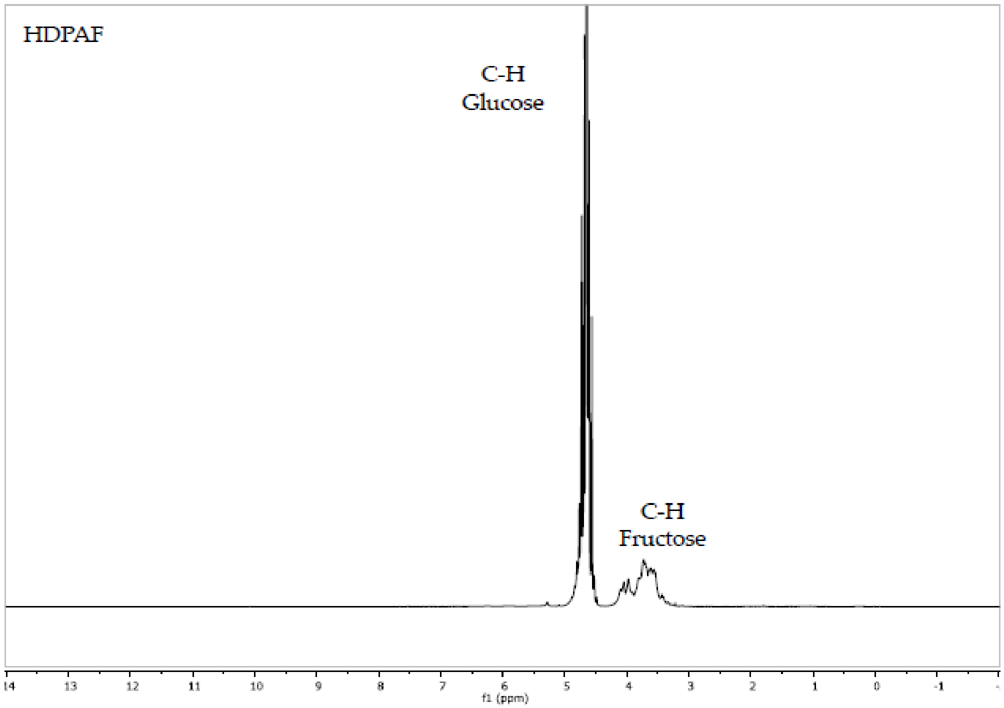
HDPAF
C-H
Glucose
C-H
Fructose
f1 (ppm)

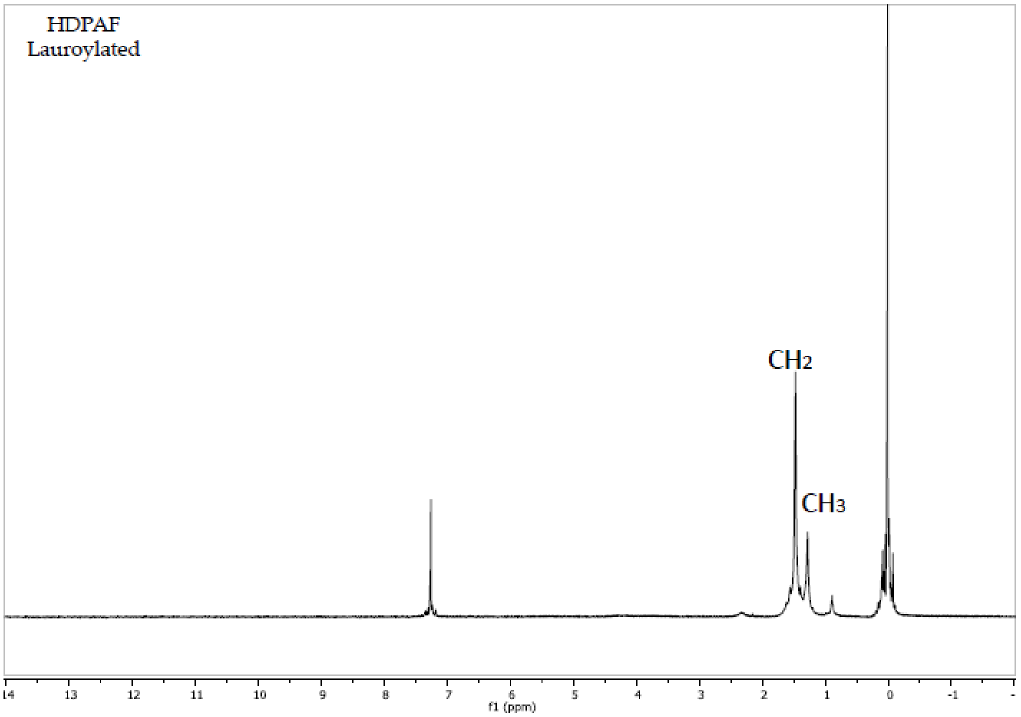
HDPAF
Lauroylated
CH2
CH3
14 13 12 11 10 9 8 7 6 5 4 3 2 1 0 -1
f1 (ppm)

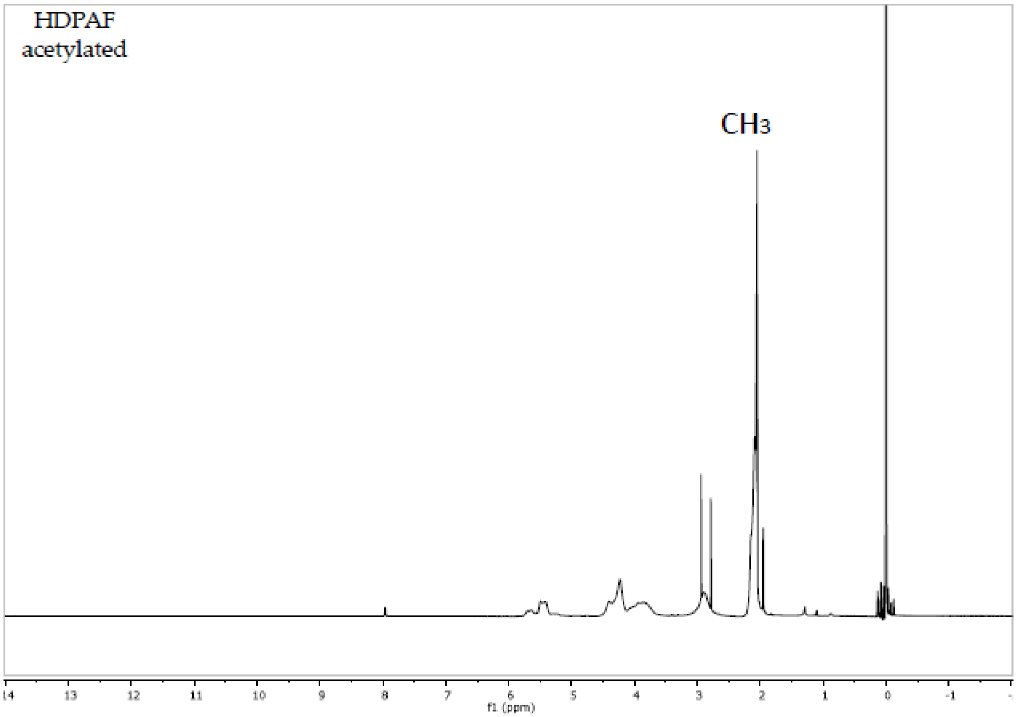
HDPAF
acetylated
CH3
14 13 12 11 10 9 8 7 6 5 4 3 2 1 0 -1
f1 (ppm)

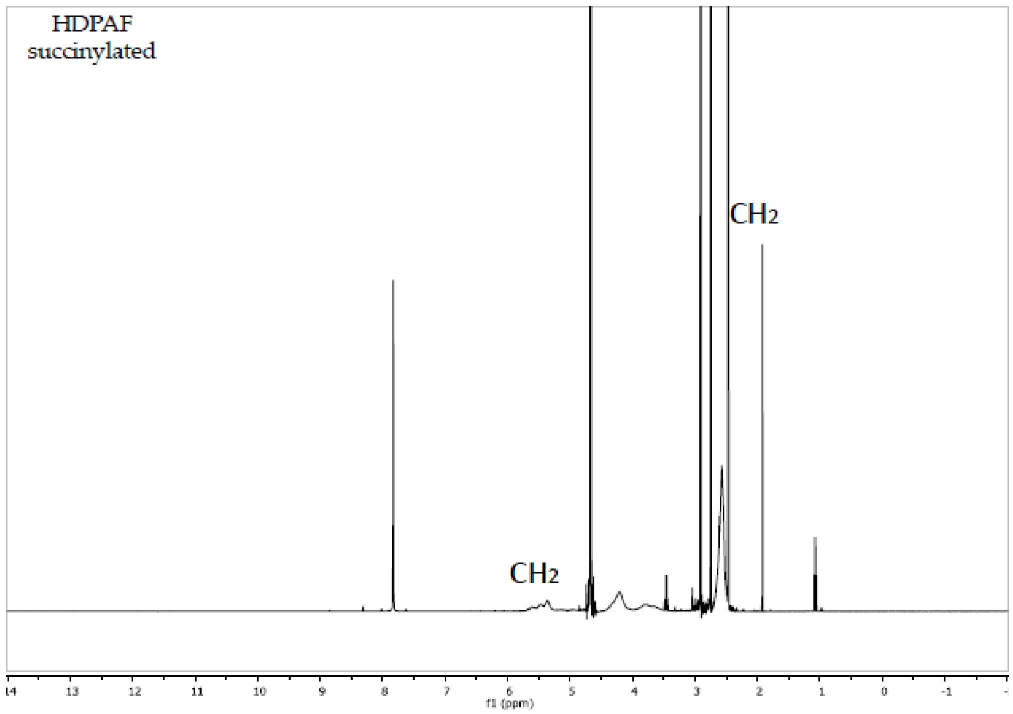
HDPAF
succinylated
CH2
CH2
14 13 12 11 10 9 8 7 6 5 4 3 2 1 0 -1
f1 (ppm)
